# Use of fluorescent nanoparticles to investigate nutrient acquisition by developing *Eimeria maxima* macrogametocytes

**DOI:** 10.1038/srep29030

**Published:** 2016-06-29

**Authors:** Sonja Frölich, Michael Wallach

**Affiliations:** 1 Children’s Medical Research Institute, Genome Integrity Group, Westmead, NSW, Australia; 2 University of Sydney, Sydney Medical School, Sydney, NSW, Australia; 3 School of Life Sciences, University of Technology Sydney, PO Box 123, Broadway, NSW, 2007, Australia

## Abstract

The enteric disease coccidiosis, caused by the unicellular parasite *Eimeria*, is a major and reoccurring problem for the poultry industry. While the molecular machinery driving host cell invasion and oocyst wall formation has been well documented in *Eimeria*, relatively little is known about the host cell modifications which lead to acquisition of nutrients and parasite growth. In order to understand the mechanism(s) by which nutrients are acquired by developing intracellular gametocytes and oocysts, we have performed uptake experiments using polystyrene nanoparticles (NPs) of 40 nm and 100 nm in size, as model NPs typical of organic macromolecules. Cytochalasin D and nocodazole were used to inhibit, respectively, the polymerization of the actin and microtubules. The results indicated that NPs entered the parasite at all stages of macrogametocyte development and early oocyst maturation via an active energy dependent process. Interestingly, the smaller NPs were found throughout the parasite cytoplasm, while the larger NPs were mainly localised to the lumen of large type 1 wall forming body organelles. NP uptake was reduced after microfilament disruption and treatment with nocodazole. These observations suggest that *E. maxima* parasites utilize at least 2 or more uptake pathways to internalize exogenous material during the sexual stages of development.

*Eimeria* spp. are obligate intracellular parasites that cause the disease coccidiosis, a major and recurring problem for livestock productivity and food security^1^,^2^,^3^. Infection of the chicken host is initiated by the ingestion of oocysts shed in the faeces. Once inside the new host, the *Eimeria maxima* parasites reach, invade, and replicate inside enterocytes lining the intestine. In studies on the asexual stages of *Eimeria*, it was found that the parasitophorous vacuole (PV) surrounding the parasite serves as a molecular sieve for acquisition of solutes, keeping the parasite’s osmotic balance in check^4^. As it develops, the *Eimeria* parasite eventually differentiates into the sexual stages. The mature male (micro-) and female (macro-) gametocytes undergo fertilisation forming an oocyst, the transmissible form of the parasite, which is released into the external environment with the faeces. The excreted oocysts of *Eimeria* are covered by a waxy cyst wall that protects the parasite against dehydration, chemical and mechanical damage allowing it to survive for extended periods of time in the harsh external environment. The cyst wall not only provides the physical barrier between the parasite and the outside environment, it also enables the development of highly infectious sporocysts, which harbour invasive sporozoites.

Discoveries made with *Eimeria* in chickens have provided valuable insights into the mechanisms of oocyst wall formation and are known to involve incorporation of tyrosine-rich precursor glycoproteins, EmGam230, EmGam82 and EmGam56 glycoproteins, synthesised by the female gametocytes^5^,^6^,^7^,^8^,^9^. The cross-linking of tyrosine residues forms a rigid matrix contributing to the robustness and impermeability of the oocyst wall. Furthermore, recent studies examining the process of oocyst wall formation in *Toxoplasma* and *Eimeria* have reported that in addition to tyrosine-rich proteins, oocyst walls of these parasites contain β-1, 3-glucan, as well as neutral and polar lipids^10^,^11^,^12^,^13^,^14^,^15^,^16^,^17^. These molecules also contribute to impermeability and rigidity of the oocyst wall. However, exactly how the parasite assembles these molecules into an impervious oocyst wall, while maintaining the ability to acquire nutrients needed for development into an infectious cyst form, remains an open question. We therefore carried out a study on the process of nutrient uptake during sexual stage parasite development.

Early studies of the encapsulation process in *E. maxima*-infected enterocytes *in situ* suggested that surface membranes of developing macrogametocytes may contain micropores^18^. These membranous structures were proposed to play a role in nutrient intake. However, since the first description of these structures, little work has been done to validate their role and explain the mechanisms by which nutrients transverse the plasma membrane and reach intracellular compartments.

In this paper, we investigated interactions of developing *E. maxima* sexual stages with negatively charged carboxylated-modified polystyrene nanoparticles (NPs) of 40 nm and 100 nm in size, as representatives of typical endocytotic cargoes widely used to study cell biology of uptake mechanisms^19^,^20^,^21^,^22^,^23^. The uptake of the model polystyrene NPs was investigated in the presence and absence of pharmacological inhibitors of different aspects of endocytosis. The inhibitors used in this study were nocodazole, a microtubule-disrupting agent, and cytochalasin D, an actin-disrupting agent. The subcellular fate and localisation of the NPs entering gametocytes were analysed by high-resolution laser scanning confocal microscopy and were analysed in comparison with control parasites grown in the absence of pharmacological inhibitors of endocytosis. The results reported here indicate that there are at least two pathways by which nanoparticles can gain access to the intracellular parasite, and that nutrient acquisition requires an intact and active cytoskeleton.

## Results

### Autofluorescence increases over time in developing *E. maxima* macrogametocytes, which is indicative of enzyme-mediated formation of dityrosine bonds

**Figure 1:**
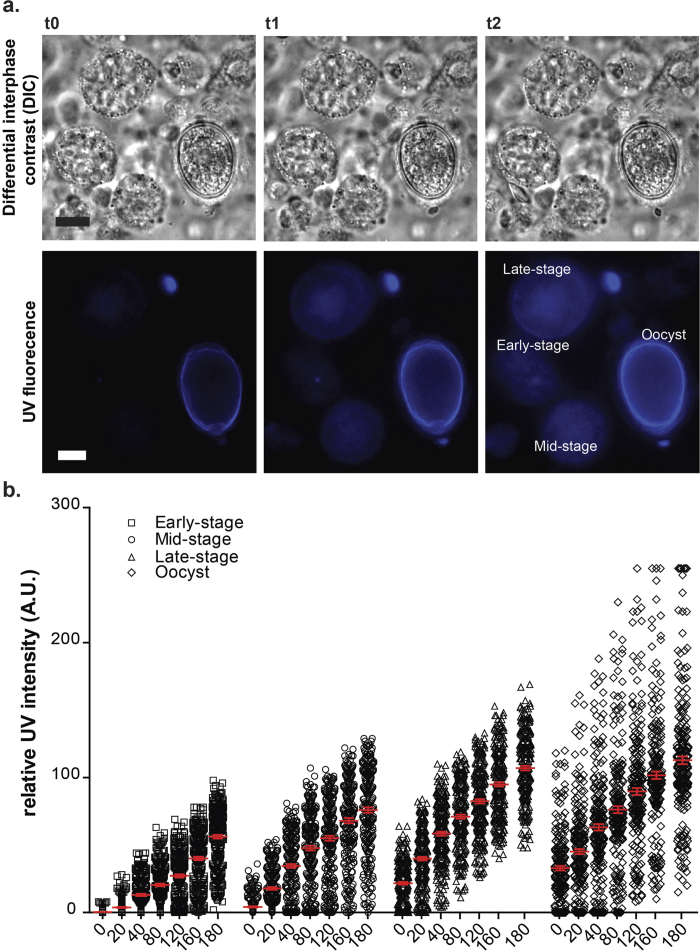
Live cell filming of freshly harvested gametocytes. (**a**) Micrographs of three time-points from a 3 hour time-lapse sequence of freshly extracted sexual stages that were acquired every 10 minutes using a combination of differential interference contrast DIC (top) and fluorescence microscopy (bottom). The cells were exposed to UV light (ex: 385 nm) to reveal the distribution of autofluorescent proteins in the macrogametocytes at early-, mid-, and late-stages of development, and in forming tissue oocysts. Note the presence of autofluorescing material in the wall of tissue oocyst at t0. As the sequence progresses, the macrogametocytes begin to exhibit blue autofluorescence (t1), at a different rate, which initially appears concentrated in the central cytoplasm, and spreads outwards to cover the entire macrogametocyte (t2). (**b**) Increase in blue autofluorescence intensity over time in live sexual stages. Autofluoresence intensities were measured by a monochromatic detector, CCD camera and ex: 385 nm. High resolution temporal information and relative intensity were recorded and time points 0, 20, 40, 80, 120, 160 and 180 minutes plotted. Scale bar = 5 μm. See also Movie 1 for a complete time-lapse experiment.

We have investigated the viability of freshly extracted sexual stages by observing and measuring changes in blue autofluorescence over time using a Nikon Ti microscope equipped with the automatic focus correction system (PFS), a LED-based epi-fluorescence illuminator and a high sensitivity cooled (CCD) camera, which allows capture of clear, high contrast images of low light fluorescence signals. Illustrated in Fig. 1a are several micrographs of freshly extracted sexual stages showing high resolution temporal information acquired on a wide-field Nikon Ti microscope over a 3 hour time-lapse sequence. It can be seen that the freshly prepared suspension is a mixture of host cells and gametocytes at different phases of sexual development, including early-, mid- and late-stages of growth and development, as well as unsporulated tissue oocysts. We monitored extracted parasites for signs of poor cellular health (i.e. cytoplasmic blebs, swelling, excessive vacuole formation, and decline in cellular activity) and observed that the parasites appeared healthy and displayed a slow and gradual increase in size, consistent with our previous report. Using an ultraviolet filter cube (ex 385 nm), we recorded blue autofluorescence emitted by the parasites over a 3 hour period. The tissue cysts in the parasite suspension were detected by the presence of an intensely fluorescent ring surrounding the egg shaped parasite (Fig. 1a, “Oocyst”), which is typical for this life cycle stage. In contrast, autofluorescence in macrogametocytes in the same field of view became visible at a later time point (t1, 80 minutes after). In these growth phases, the blue autofluorescence appeared more concentrated in the central cytoplasm with some areas in the periphery of the macrogametocyte appearing dark (Fig. 1a, “Late stage” at t1). As the sequence progressed, the blue autofluorescence then spread to cover the entire cell (Fig. 1a, “Late stage” at t2).

A comparison of the relative intensities of fluorescence in different stages of sexual development is shown in Fig. 1b. These data were obtained from measurements using a sensitive CCD camera. In that way, not only the relative intensities of the population as a whole, but also the distributions of fluorescence from the individual cells were compared. It can be seen that sexual growth phases displayed very different blue fluorescence intensities due to varying amounts of the protein dityrosine. For example, tissue oocysts displayed the highest relative fluorescent intensity reaching a maximum value of 255, whereas the maximum intensity in the early-stage microgametocyte reached 98, 1.5 times less. The changes in parasite blue autofluorescence over time are shown in Movie 1.

In order to be certain that the parasites were alive using this chamber configuration, we set up a control viability experiment in parallel to the autofluorescent test in this experiment. The parasites were maintained under the standard growth conditions for the duration of the 3 hour incubation period (culture medium, humidity, temperature, chamber configuration) used above, and we tested them for viability by the trypan blue exclusion test. Based on cell counts, more than 85 percent of the parasites excluded the toxic dye following 3 h of incubation (not shown).

### Developing macrogametocytes and oocysts take up and internalize nanoparticles *in vitro*

**Figure 2:**
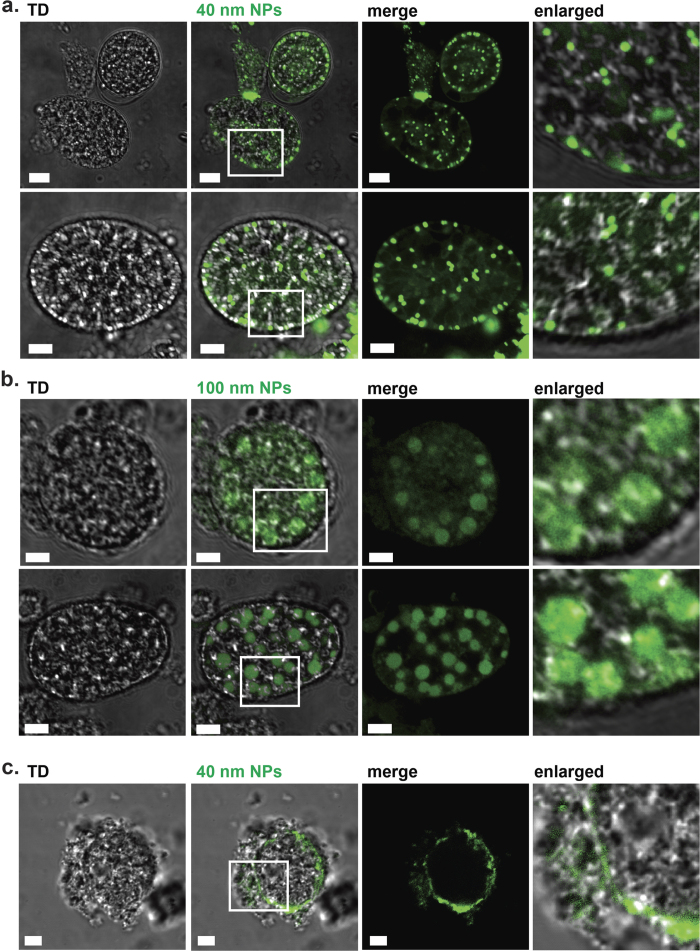
Nanoparticle uptake by freshly harvested gametocytes. Freshly harvested sexual stages incubated with 40 nm (**a**) or 100 nm (**b**) particles for 1 h at 37 °C. LSM micrographs demonstrate beads of 100 nm, but not of 40 nm localise to the type 1 wall forming body vesicles. Inserts represent close up intracellular events of 40 nm and 100 nm particles in macrogametocytes. (**c**) Extracted sexual stages exposed to nanoparticles for 1 h at 4  °C. Note the absence of intracellular events and accumulation of the particles (green) at the surface of the parasite surrounded by the host cell. Scale bars correspond to 5 μm.

We made use of green FluoSpheres^®^ (Life technologies, Australia) with nominal sizes of 40 and 100 nm in order to assess the amount and intensity of cellular uptake and internalization of the beads into gametocytes at different time points of sexual development. We focused the work on these nanoparticle sizes, as being representative of a range of endocytotic cargoes such as lipoproteins (LDL and VLDL) which are below the limit of specialised phagocytosis. Examination of gametocytes by high-resolution confocal microscopy revealed that the internalized particles of 40 nm were distributed throughout the parasites (Fig. 2a). More specifically, the green 40 nm particles were confined to discrete cytoplasmic vesicle-like regions approximately 0.5–1 μm in diameter situated both on the surface and distributed throughout the macrogametocyte (Fig. 2a, top panels). In addition, diffusely fluorescing filamentous structures appeared to be associated with the strongly fluorescing aggregates (Fig. 2a, enlarged). In early stage oocysts, we observed that the fluorescent signal localised to the parasite’s surface (Fig. 2a, bottom panels); however, even in those samples internalised particles were observed (Fig. 2a, bottom panel enlarged).

Figure 2b shows that the 100 nm particles were also taken up and internalized by the freshly extracted macrogametocytes and early stage oocysts. In that case, the green 100 nm particles were localised predominantly to large type 1 wall forming body (WFB) vesicles (Fig. 2b, enlarged panels), suggesting that the cytosolic streaming of internalized particles is size dependent.

In order to determine whether nanoparticle uptake is an energy dependent process, uptake experiments were performed at 4 °C or in the presence of sodium azide. Confocal micrographs (Fig. 2c) show that exposure to 40 nm particles at 4 °C resulted in a very strong inhibition of uptake. Similar results were obtained with the ATPase inhibitor sodium azide (not shown), and freeze-thawed gametocytes, which do not have intact membranes and were used as a negative control (not shown).

### Effect of inhibitors of F-actin and microtubules on the uptake of nanoparticles

**Figure 3:**
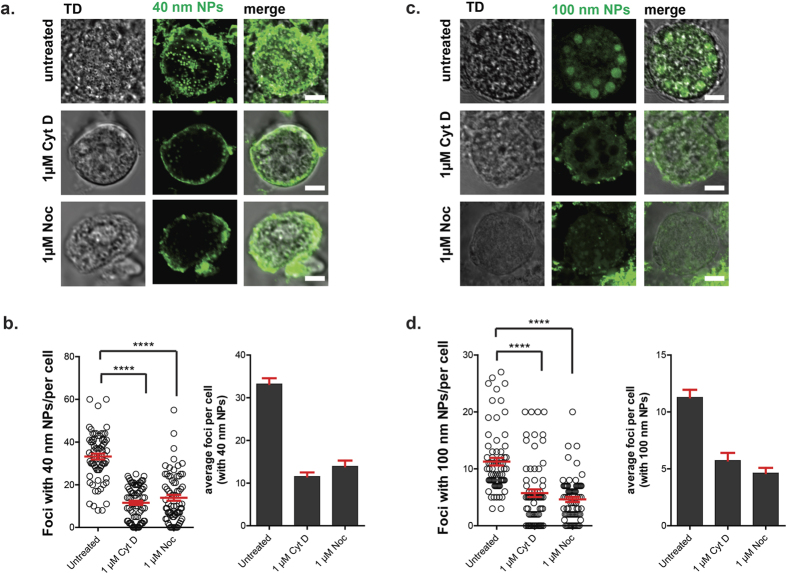
Effect of cytoskeletal inhibitors on uptake of nanoparticles. (**a**) Fluorescent confocal 2D micrographs of fresh macrogametocytes incubated in the presence of fluorescent 40 nm nanoparticles prior to and after the addition of cytoskeletal inhibitors, cytochalsin D and nocodaloze. Intracellular 40 nm NPs are shown in green. Scale bar: 5 μm. (**b**) Quantification of foci containing intracellular 40 nm particles in cells with and without cytochalasin D and nocodazole (mean ± range, n = 3, *P* = two-tailed t-test). (**c**) Confocal micrographs showing uptake of 100 nm particles (particles: green) in untreated and drug-treated macrogametocytes. Scale bar: 5 μm. (d) Quantification of foci containing intracellular 100 nm particles in cells with and without cytochalasin D and nocodazole. Mean ± range, n = 3, *P* = two-tailed t-test.

The cytoskeleton plays an important role in endocytosis and trafficking of endocytotic vesicles, therefore, it was of interest to examine whether differences in internalization could be revealed as a function of cytoskeleton inhibition. Parasites were pre-treated with 1 μM cytochalasin D to inhibit F-actin polymerization, and subsequently incubated with 40 nm or 100 nm nanoparticles, as described in Materials and Methods. In control cells that were not treated with inhibitors, uptake of both small (40 nm) and large (100 nm) nanoparticles was observed within only a few minutes of incubation in both mature macrogametocytes and early-stage oocysts (Fig. 3, untreated in a,b). Pre-treatment of the parasites with cytochalasin D inhibited the uptake of 40 nm beads (Fig. 3a,b), with dose of 1 μM significantly (P < 0.0001) reducing uptake, where very few foci containing fluorescent 40 nm particles were visible in the cytoplasm (Fig. 3a, “1 μM Cyt D treated” panels). Similarly, pre-treatment with 1 μM cytochalasin D also significantly inhibited the uptake of 100 nm particles (Fig. 3c,d, P < 0.0001). Although a few foci containing 100 nm particles could be seen in the cytosol of parasites treated with 1 μM cytochalasin D, the subcellular distribution differed significantly in comparison to the untreated controls.

Nocodazole was used as a tubulin inhibitor in order to investigate the role of microtubules in the internalization of small (40 nm) and large (100 nm) nanoparticles. As can be seen in Fig. 3a, exposure to nocodazole inhibited the uptake of 40 nm particles, with a significant reduction (P < 0.0001) in intracellular fluorescence observed at 1 μM (Fig. 3a, “Noc treated”). However, there did not appear to be complete inhibition of uptake of the small NPs since a few green 40 nm particles were detectable in the parasite’s cytoplasm (Fig. 3a, “1 μM Noc treated” panels).

Nocodazole was found to inhibit endocytosis of larger particles (100 nm), with a significant fraction of the nanoparticles observed outside of the cell membrane at 1 μM concentrations (Fig. 3c,d, P < 0.0001). In contrast to control cells (Fig. 3c, “untreated” panels), the intracellular distribution of the larger particles was found to be very different in nocodazole treated parasites, with none localising to large type 1 wall forming body vesicles (Fig. 3c, “1 μM Noc treated” panels”). These results suggest that both microtubules and F-actin are involved in the uptake of larger particles, as expected for macropinocytosis.

### Particles of 100 nm, but not of 40 nm, accumulate in the type 1 wall forming bodies

**Figure 4:**
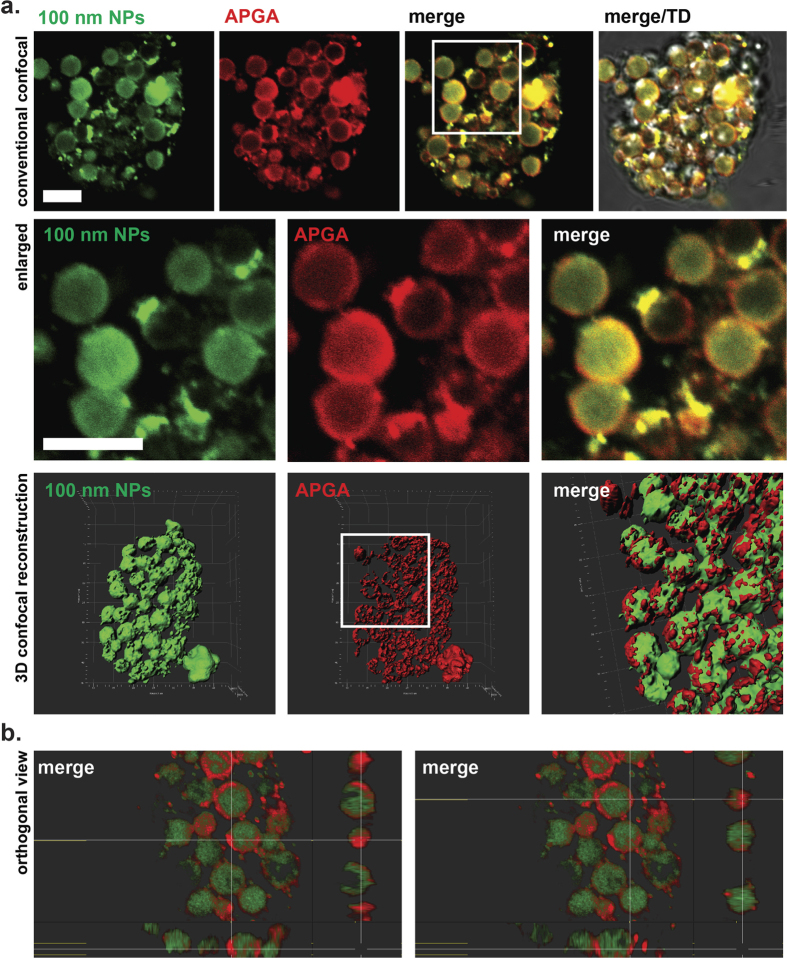
Laser scanning microscopy imaging revealed colocalisation of 100 nm particles with large type 1 wall forming bodies. (**a**) Distribution of internalized 100 nm NPs (green) and the anti-APGA antibodies (red) using conventional 2D confocal microscopy. Scale bar: 5 μm. Enlarged panels depict localisation of 100 nm beads (green) to anti-APGA positive type 1 wall forming bodies. Bottom: 3D computer reconstruction and isosurface-rendered images were generated from 5–20 confocal z-stacks of 0.5 μm thick optical cross sections. Quadrants = 5 μm. (**b**) Orthogonal cross-sections along the x axis highlight the spatial distribution of internalized 100 nm particles. The lumen of type 1 wall forming body vesicles (demarked by red antibody labelling) appears filled with numerous green 100 nm particles. Scare bar: 5 μm.

To further analyse the subcellular localization of nanoparticles, freshly prepared sexual stages were first incubated with 100 nm or 40 nm particles for 1 h at 37 °C followed by fixation and incubation with red-fluorescent anti-Affinity Purified Gametocyte Antigen (APGA) antibodies, which are specific to antigens localised to the type 1 and type 2 wall forming body organelles. In addition, red-fluorescent Evans blue dye, which selectively binds to the type 1 wall forming body vesicles, was also used in the experiment. 3D confocal microscopy was used to collect high resolution spatial information where it was found that the anti-APGA antibodies colocalised with the nanoparticles of 100 nm (Fig. 4a). Figure 4b clearly shows that 100 nm beads consistently migrated to the lumen of the large type 1 wall forming body vesicles, highlighted by the red anti-APGA antibodies. Orthogonal cross-sections along the Z axis of the double-stained parasites confirm this localization (Fig. 4b).

**Figure 5:**
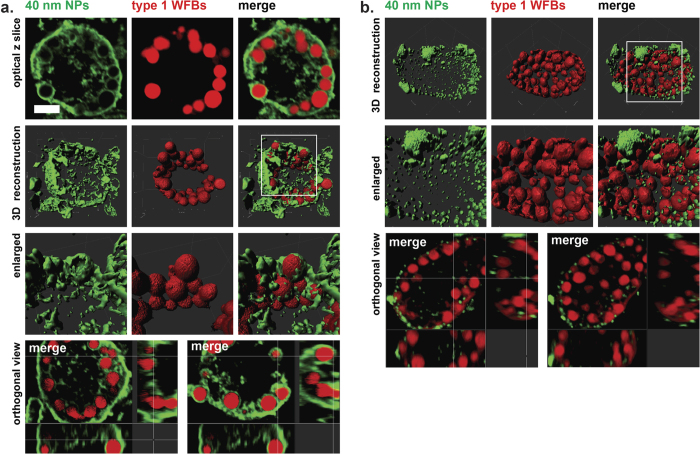
High resolution spatial imaging of green fluorescent 40 nm particles inside the mature macrogametocyte and forming tissue cysts counter-stained with Evans blue. (**a**) 3D projection and corresponding isosurface-rendered images of the mature microgametocytes were generated from confocal z-stacks of 0.5 μm thick optical cross sections. The type 1 wall forming body vesicles are highlighted by Evans blue stain (red), as described in Materials and Methods. Cellular distribution of 40 nm nanoparticles is shown in green. Enlarged box shows magnified cellular compartment illustrating intracellular distribution of 40 nm particles. Quadrants = 10 μm. (**b**) Three-dimensional distribution of 40 nm (green) beads in an early-stage oocyst labelled with Evans blue (red). Cellular distribution of 40 nm nanoparticles is shown in green. Close up top view of the region depicted in (**b**) showing 40 nm particles (green) localised to the cell surface and within the cytoplasm, with nanoparticles accumulated in small aggregates. Orthogonal view of an image in (**b**) showing the luminal to basal surface along the Z axis (z slicing 0.5 μm). Micrographs represent the intracellular distribution of 40 nm particles. Quadrants = 10 μm. See Movies 2 and 3 for 3D spatial distribution of 40 nm NPs in mature macrogametocytes.

From Fig. 5, it can be seen that the smaller 40 nm nanoparticles were distributed throughout the cytoplasm of both mature macrogametocytes (Fig. 5a) as well as the early-stage oocysts (Fig. 5b) but did not co-localize with type 1 wall forming body (Fig. 5a, merge panels in “optical z slice”). To determine their intracellular localisation we counter-stained the parasites with the Evans blue dye. Using 3D confocal microscopy and orthogonal sections (Fig. 5b), the cellular distribution of the green 40 nm beads was compared to that of type 1 wall forming bodies. Z series images of parasites clearly showed a cytoplasmic distribution of the 40 nm nanoparticles in small vesicles not associated with the type 1 wall forming bodies (Fig. 5a,b “enlarged” panels).

## Discussion

The aim of the present study was to gain an understanding of how *E. maxima* sexual stages acquire nutrients necessary for gametocyte development and oocyst biogenesis. In order to perform this study we employed fluorescent nanoparticles and studied their uptake and internalization in freshly extracted gametocytes and early stage oocysts maintained live using *in vitro* cell culture. We chose to use 40 and 100 nm nanoparticles since they are representative of nutrients such as organic macromolecules (i.e. carbohydrates, LDLs, HDLs, peptides). Visualization of the fluorescent nanoparticles was performed using 3D confocal imaging technologies. Finally, the uptake of the nanoparticles and their internalization was observed in the presence and absence of chemical inhibitors of endocytosis.

The first part of the work was focused on the viability of the gametocytes during *in vitro* culture to ensure that the parasites were alive and metabolically active. The parasites were tested for trypan blue exclusion, where it was found that over 85% of the parasites excluded the dye showing that they are indeed alive. Secondly, time lapse studies on freshly extracted *E. maxima* gametocytes was performed over a 3 hour period and UV autofluorescence was measured as an indication of the active processing and cross-linking of tyrosine rich wall forming body proteins and oocyst wall development. It was found that there was a strong increase in fluorescence during the incubation period eventually reaching a plateau (see Fig. 1). These results together with previous studies showing that isolated gametocytes are metabolically active using [S^35^] methionine labelling^12^,^24^,^25^, demonstrated that for at least 3 hours the parasites are synthesizing proteins, enzymatically active and viable.

We next went on to test the uptake of the 40 and 100 nm nanoparticles and study their subcellular localisation. 2D micrographs of *E. maxima* sexual stages pre-incubated with 40 nm particles were examined and it was found that the cytoplasm of both macrogametocytes and oocysts fluoresced green. Interestingly, the 40 nm particles localised to small cytoplasmic vesicles, which we predict represent endosome-like bodies in which extracellular material is broken down and utilized by the parasite for nutrition. In contrast, studies using the larger nanoparticles of 100 nm showed a very different fluorescent profile and were specifically associated with the type 1 wall forming bodies. This surprising finding indicates that the type 1 wall forming bodies play a role in both oocyst wall formation as well as in incorporating and perhaps processing extracellular molecules. What is clear from these results is that the 40 and 100 nm nanoparticles are taken up through 2 different pathways of endocytosis dependent upon the size of the particles.

3D confocal imaging technology was carried out in order to determine the precise spatial localisation of the beads within the isolated sexual stages. We found that the 100 nm beads were found in the lumen of the type 1 wall forming bodies while the 40 nm beads were found in the cytoplasm with no apparent association with the type 1 or 2 wall forming bodies. Thus, we concluded that these two pathways are indeed distinct from one another and that the lumen of type 1 wall forming bodies may act as a large reservoir of extracellular macromolecules.

In a distantly related cyst forming parasite, *Giardia*, it was shown that the developing parasites gain access to nutrients via pinocytosis and specialised endocytic vesicles termed peripheral vesicles (PVs)^26^,^27^,^28^. During the encystation process the parasites also induce neogenesis of Golgi-like organelles, encystation-specific vesicles (ESVs), for regulated secretion of cyst wall material. It was shown that both PV and ESV organelle systems intersect physically and functionally at the endoplasmic reticulum (ER) which serves both catabolic and anabolic functions. Whether the type 1 wall forming bodies and additional small vesicles identified in the present study in *Eimeria* pre-encysting stages serve similar functions during *E. maxima* oocyst biogenesis remains to be investigated.

The cytoskeleton is known to play an essential role in endocytosis and nutrient uptake^29^. It can support the processes of invagination of a membrane segment into the cytoplasm, scission of the new vesicle from the plasma membrane, and movement of the vesicles away from the surface membrane. Recently, we showed how the actin cytoskeleton interacts with the type 1 wall forming bodies during oocyst biogenesis, presumably to transport them to the surface of the encapsulating gametocytes for incorporation into the outer oocyst wall^30^. Our finding of the colocalisation of the 100 nm particles and the type 1 wall forming body vesicles is an indication that a cytoskeleton-mediated pathway is involved in the uptake of the 100 nm NPs by developing sexual stages. In support of this hypothesis, we found that the uptake of both 40 and 100 nm nanoparticles could be blocked by cytochlasin D, an inhibitor of actin polymerization. This reduced or total block in uptake of NPs was associated with their accumulation at the cell surface in the presence of the drug. This effect may be due to alterations in the actin cytoskeleton that prevented the formation of endocytotic vesicles at the cell surface.

Very little is known about the PV membrane of *Eimeria* sexual stages mainly due to difficulties in producing gametocytes and oocysts using *in vitro* culture systems despite recent progress. There is only one study suggesting the presence of pores on the surface of developing *E. maxima* oocysts^18^. An early electron microscopy (EM) study of *in situ E. maxima* gametocytes at different stages of encystation described the existence of large numbers of micropores; cup-shaped invaginations of the plasma membrane associated with an elaborate tubular network. The authors suggested that the pores could facilitate transport of materials between the parasite and its host cell. Our results support the presence of pores in the PV membrane of the macrogametocyte and in the early stages of oocyst development.

We found that the inhibition of 40 and 100 nm NP uptake also occurred in the presence of the microtubule inhibitor, nocodazole. This suggests that in the sexual stages of development both actin and tubulin mediate the endocytotic processes. Further work is needed using additional inhibitors such as wortmannin, chlorprozamine, filipin or nystatin to determine if receptors such as clathrin and caveole are also involved in the uptake of exogenous material by developing gametocytes.

Finally, the work described in this paper using cytoskeletal inhibitors to reduce or prevent the uptake of nanoparticles, may lead to a better understanding of the molecular mechanisms governing membrane transport of nutrients in *Eimeria*-infected enterocytes. This may also help to provide insight into possible new routes for delivering cytotoxic agents into the developing intracellular parasite.

## Materials and Methods

### Experimental animals and parasite production

All procedures were carried out in accordance with guidelines approved by the Animal Care and Ethics Committee at the University of Technology, Sydney (ACEC# 2013-099A). The Houghton strain of *E. maxima* used throughout these experiments was originally provided by Martin Shirley (Institute for Animal Health, Compton, Newbury, Berkshire, United Kingdom). The oocysts were periodically passaged in 3–7 week old light breed Australorp chickens (Barter and Sons Hatchery, Luddenham, Australia) using established methods^31^. Oocysts were harvested from the faeces, sporulated *in vitro* at 30 °C in an incubator and isolated by salt floatation and bleach treatment (2%, Milton solution). Gametocytes were prepared using techniques published previously^24^. Briefly, chickens were infected at 4 weeks of age with freshly propagated *E. maxima* oocysts. They were sacrificed 134–136 hours post infection and their intestines immediately removed and washed with pre-warmed (37 °C) PBS (pH 7). The washed intestines were slit open lengthwise and the mucosa scraped gently to collect the infected epithelia. The collected material, rich in gametocytes, was placed on top of a 17 μm polymon filter and washed with pre-warmed (37 °C) SAC buffer (170 mM NaCl, 10 mM Tris-HCl pH 7, 10 mM glucose, 5 mM CaCl_2_, 1 mM PMSF, 1 mg/ml BSA). The material on the filter was discarded and the flowthrough, containing the gametocytes, was filtered through a 10 μm polymon filter. The gametocytes which accumulated on the filter were collected by centrifugation and enumerated.

Viability of freshly harvested gametocytes was assessed using the trypan blue exclusion test, as previously described^32^. Briefly, 100 μl of freshly prepared parasites were added to a microfuge containing 100 μl of 0.4% Trypan blue solution (Life technologies). The suspension was gently mixed by tapping and viable cell density determined using a haematocytometer. The percentage of non-viable parasites was determined as the ratio of dead parasites (taking up the trypan blue dye) versus the entire parasite population X 100. All measurements were done in triplicate, and at least three independent experiments were carried out (data not shown).

The quality of harvested gametocytes was then assessed for the presence of wall forming bodies by cytochemical stains, western blotting and immunostaining using wall forming body-specific antibodies, as described below. Subsequently, gametocytes were either immediately used unfixed in uptake experiments, or cells were fixed for 10 minutes directly in SAC buffer (pH 7), by 2% paraformaldehyde.

### Wide-field time-lapse microscopy of harvested *E. maxima* gametocytes

Live-cell imaging and sequential measurements of UV intensity changes of freshly harvested and freeze-thawed gametocytes, used as a control for these experiments, was performed in two spatial dimensions using wide-field techniques and time-lapse video microscopy, as previously described^12^. Briefly, freshly harvested gametocytes (2 × 10^6^) were resuspended in 1 ml of SAC buffer (pH 7) and a drop of gametocyte suspension was placed on a round 35-mm glass-bottom dish. The cells were left to settle for 15 min in a microscope heated chamber. The chamber was pre-warmed to 37 °C and humidified (5% CO_2_) before imaging on an automated epifluorescence Nikon Eclipse Ti-U inverted microscope (Nikon, Tokyo, Japan) equipped with a 60 X oil objective lens (Plan Apo NA 1.4 aperture) and the Perfect Focus System™ for continuous maintenance of focus. Differential interference (DIC) and the corresponding UV images (excitation: 470/40 nm, emission: 525/50 nm) were acquired every 10 minutes over a 3 h period with a high-speed charge-coupled device (CCD) camera. Each movie was then exported in a separate folder from NIS Elements acquisition software as uncompressed TIFF files and accompanying Excel spreadsheets analysed using GraphPad prism.

### Uptake experiments with nanoparticles

Green fluorescent (excitation/emission wavelengths: 505/515) polystyrene carboxylate nanoparticles (FluoSpheres^®^, Life technologies) were used without further modifications. NP sizes used in this study were 40 nm and 100 nm. Stock solutions were stored at 4 °C and used less than 1 year after purchase, as recommended by the manufacturer to ensure their stability. Immediately prior to the experiments with parasites, NP dispersions were prepared by diluting the concentrated stock in SAC medium (1:1000), with or without drugs, and sonicating for 15 min in a water bath in order to avoid aggregation. Before sampling, NPs were adjusted to a concentration of 20 μg/mL in SAC and further mixed by vortexing, as recommended by the manufacturer. The nanoparticles were incubated with freshly extracted gametocytes in SAC medium for 1 h at 37 °C. Removal of attached beads was accomplished by washing the parasite extract three times (5 min, 1,000 × g) in PBS, then fixed for 20 min with 2% paraformaldehyde. As a control for these experiments, freeze-thawed parasites, which do not have intact membranes based on viability staining, were also incubated with the nanoparticles and processed as above. Uptake experiments were also carried out at 4 °C to test the effect of temperature on internalization. Sodium azide was used as ATPase inhibitor. Processed parasites were then mounted in Vectashield^®^ (Vector Laboratories, Burlingame, CA, USA) prior to analysis using a laser scanning confocal microscope, as described below. For particle uptake analysis, >25 cells were quantified for each experimental replicate. Statistical analysis was performed using Prism 6 (GraphPad Software, San Diego, CA, USA). Data are expressed as means ± s.e.m. unless otherwise stated. Data from two groups were compared using Mann-Whitney 2-tailed *t-*test for paired samples. Values of *P* ≤ 0.05 were considered statistically significant.

### Inhibition of endocytosis

Freshly extracted sexual stages were pre-incubated with widely used inhibitors of endocytosis; cytochalasin D (0.01–10 μM) for 30 min at 37 °C to disrupt actin cytoskeleton^33^,^34^, or nocodazole (0.01–10 μM) to disrupt microtubules^35^,^36^,^37^. Consecutively, nanoparticles of 40 or 100 nm in diameter were added and incubation continued for 1 h, at 37 °C. Subsequently, exposed parasites were washed to ensure particle removal and analysed by confocal laser scanning microscopy. Negative controls, i.e. cells without the presence of drugs or nanoparticles and those treated with the vehicle (DMSO) were also carried out. All inhibitors were obtained from Sigma Aldrich.

### Laser scanning confocal microscopy of fixed macrogametocytes

A Nikon A1 confocal laser scanning microscope was used to capture high-resolution images of the intracellular environment layer by layer and the sub-cellular localization of the fluorescent nanoparticles. Following the exposure to nanoparticles, the extracted parasites were washed and co-incubated with the wall forming body-specific fluorescent dye markers using published methods. Briefly, parasites were fixed with 2% paraformaldehyde for 10 minutes at room temperature. The parasites were then washed three times with PBS, then permeabilised for 10 min with 0.1% Triton X-100 (v/v) and then washed again with PBS. Non-specific binding was blocked by incubation for 1 h in PBS containing 2% (w/v) BSA prior to incubation in the presence of a mouse polyclonal IgG to Affinity Purified Gametocyte Antigens (APGA; 1:250). The primary antibodies were detected with AlexaFluor594 conjugated goat anti-mouse secondary antibody (1:500; Life technologies). Alternatively, large type 1 wall forming bodies were detected using Evans blue dye, as described previously^30^,^38^,^39^. Both primary and secondary antibodies were diluted in 2% (w/v) BSA in PBS and incubated for 1 hr at room temperature. Finally, cells were washed, mounted in Vectorshield (Vector laboratories) and visualised.

Image acquisition was performed with an inverted Nikon A1 scanning laser microscope (Nikon) equipped with 488, 561 and 637 laser excitation sources. The experiments were performed using 63X and 100X (1.4 NA) immersion oil lens. The gain and offset for the different channels were kept constant during the experiments. The three-dimensional (3D) structure of the cells was reconstructed from corresponding confocal images using Imaris software (v 8.0, Bitplane).

## Additional Information

**How to cite this article**: Frölich, S. and Wallach, M. Use of fluorescent nanoparticles to investigate nutrient acquisition by developing *Eimeria maxima* macrogametocytes. *Sci. Rep.* **6**, 29030; doi: 10.1038/srep29030 (2016).
